# The Effects of High-Intensity Interval Training on Inflammatory Cytokines in Children and Adolescents with Obesity: A Systematic Review and Meta-Analysis

**DOI:** 10.3390/metabo16010088

**Published:** 2026-01-21

**Authors:** Meng Cao, Pei Sun, Xiaodong Wang, Mengxian Zhao

**Affiliations:** 1 College of Sport, Shenzhen University, Shenzhen 518060, China; caomeng123@szu.edu.cn (M.C.); wang_xd@szu.edu.cn (X.W.); 2 Faculty of Health and Wellness, City University of Macau, Macau 999078, China; peisun@cityu.edu.mo; 3 Wangxiaodong Sports Rehabilitation Studio, Shenzhen Bao’an District, Shenzhen 518100, China

**Keywords:** interval training, inflammation, C-reactive protein, children, obesity

## Abstract

**Background**: High-intensity interval training (HIT) is a time-efficient strategy to improve metabolic health in children, but its impact on inflammatory markers is still unclear. Therefore, we conducted a meta-analysis to examine the role of HIT on pro-inflammatory cytokines including C-reactive protein (CRP), interleukin 6 (IL-6), and tumor necrosis factor-alpha (TNF-α) in children with overweight/obesity. **Methods**: A meta-analysis was conducted following PRISMA guidelines. PubMed, Web of Science, Scopus, and Embase were searched up to 31 July 2025, for studies involving children with overweight/obesity aged 6 to 18 years. Randomized controlled trials and non-randomized controlled trials with outcome measurements that included CRP, IL-6, and TNF-α were included. Random-effects models were used to aggregate a mean effect size (ES) with 95% confidence intervals (CI), and potential moderators were explored. **Results**: In total, 768 participants from 15 studies were included. HIT significantly improved CRP (574 participants, 13 studies, SMD = −0.63, 95% CI: −1.02 to −0.24, *p* < 0.01) when compared to control group/pre-intervention. There were no significant effects on IL-6 and TNF-α, and no differences when compared to moderate-intensity training. Subgroup analyses indicated greater effectiveness in intervention duration, work-and-rest ratio, and work time were the significant moderators (*p* < 0.05). **Conclusions**: High-intensity interval training is effective for reducing CRP levels in children with obesity. Intervention duration, work-and-rest ratio, and work time can affect the intervention effects of HIT.

## 1. Introduction

The global incidence of pediatric obesity has risen markedly, constituting a major challenge for public health [1]. Excess weight and obesity in childhood predispose individuals to conditions such as hypertension, dyslipidemia, and insulin resistance later in life. Compounding this risk, obesity fosters a chronic, low-grade inflammatory state that significantly elevates the chances of developing cardiovascular and metabolic diseases [2]. Research indicates that the inflammatory response associated with obesity correlates with changes in plasma levels of C-reactive protein (CRP), interleukin-6 (IL-6), and other inflammatory biomarkers [3]. In individuals with obesity, both hypertrophic adipocytes and immune cells that infiltrate adipose tissue undergo phenotypic and functional alterations. These changes shift their secretion profile from anti-inflammatory cytokines to pro-inflammatory adipokines and cytokines, ultimately triggering systemic inflammation [4]. CRP serves as one of the most sensitive indicators of inflammation, with its concentration rising in response to increasing inflammatory activity [5]. Studies have demonstrated that a reduction in CRP levels correlates with a lower risk of cardiovascular events across both lean and obese populations [6,7]. Elevated CRP levels contribute to heightened insulin resistance and increased lipogenesis, thereby creating a vicious cycle that worsens obesity [8]. On the other hand, IL-6 is a pleiotropic cytokine that exerts diverse physiological effects. Excessive production of IL-6 can trigger systemic inflammatory responses and promote the elevation of various cytokines. Individuals with elevated levels of both CRP and IL-6 face a heightened risk of mortality from cardiovascular diseases—this association remains independent of factors such as age, gender, body mass index (BMI), or medical history.

Engaging in regular physical activity serves as an effective approach to preventing excessive weight gain and enhancing cardiometabolic well-being. The World Health Organization (WHO) recommends that children and adolescents between the ages of 5 and 17 engage in a minimum of 60 min of moderate to vigorous physical activity daily [9]. The anti-inflammatory properties of moderate-intensity continuous training (MICT) have been demonstrated in meta-analyses of patients with chronic diseases [10]. However, factors such as limited time availability, insufficient motivation, poor compliance, and difficulty in sustaining exercise-induced long-term physiological adaptations have been recognized as obstacles that hinder children and adolescents from participating in moderate-intensity continuous training (MICT) [11]. High-intensity interval training (HIT)—which consists of short bursts of vigorous activity interspersed with rest—has proven to be an effective and efficient alternative for promoting weight loss and boosting cardiometabolic well-being [12]. There is substantial evidence supporting the effectiveness of HIT in enhancing body composition, boosting cardiorespiratory fitness, and regulating glucose and lipid metabolic among overweight or obese youth [13,14,15]. Furthermore, meta-analytic evidence regarding the impact of exercise on inflammatory cytokines in children and adolescents with overweight/obesity remains inconsistent. For example, one study by Li et al. demonstrated that physical activity resulted in decreased CRP levels in this population [16]. In contrast, Khalafi’s study found no significant effect in healthy children [17]. In addition, a meta-analysis focusing specifically on HIT reported improvements in tumor necrosis factor-alpha (TNF-α), leptin, and adiponectin levels among individuals with metabolic disorders, though no significant changes were observed in CRP levels [18]. However, meta-analysis of effects on regulation of inflammatory factors involved in HIT are relatively limited, especially concerning its impact on children with overweight/obesity [19,20,21,22]. Additionally, the current body of research exhibits several limitations: (1) no prior meta-analysis has specifically investigated the impact of HIT on inflammatory cytokines in obese children; (2) several recent and pivotal studies were omitted from consideration; and (3) there has been no in-depth examination of the possible explanations for the observed inconsistencies through subgroup analyses or meta-regression techniques.

Accordingly, this research employed a meta-analysis and systematic review to investigate the effects of HIT on inflammatory markers (CRP, IL-6, TNF-α) in overweight/obese children, including an evaluation of how different HIT protocols modulate these effects.

## 2. Methodology

### 2.1. Research Protocol Registry

Adherence to the PRISMA (Preferred Reporting Items for Systematic Reviews and Meta-Analyses) statement was maintained throughout this review of both randomized and non-randomized controlled trials [23]. The registered information of this study is in the International Prospective Register of Systematic Reviews (PROSPERO) with ID: CRD42021291473.

### 2.2. Data Sources and Search Strategy

**Figure 1:**
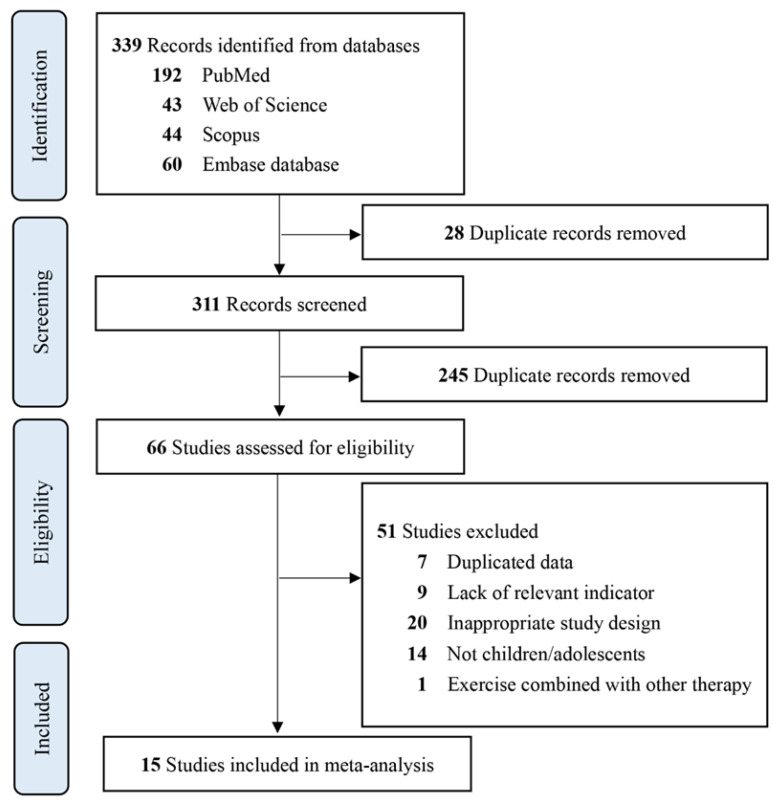
Literature screening flow diagram based on PRISMA.

Literature searches were performed across major English-language electronic databases—including Web of Science, PubMed, Scopus, and Embase—covering publications from January 2000 through July 2025. The following search terms were included and combined using the operators ‘AND’, ‘OR’, and ‘NOT’: ‘overweight’ or ‘obesity’ AND ‘high-intensity interval training’, or ‘aerobic interval training’ or ‘sprint interval’ AND ‘child*’ or ‘adolescent’ AND ‘c-reactive protein’, or ‘CRP’ or ‘Interleukin’ or ‘IL-6′ or ‘TNF-α’ or ‘inflammatory’. The details of search strategies are described in the Supplementary Materials. Initially, the title and abstract are evaluated by two independent reviewers (MC and PS). Afterwards, full-text articles that may meet the criteria are reviewed, and a determination is made on their inclusion in the study. The flow of studies during the selection process is depicted in Figure 1. Full details of the database-specific search strategies are provided in Appendix A Table A1.

### 2.3. Inclusion and Exclusion Criteria

Peer-reviewed studies were selected based on participants, interventions, comparisons, outcomes, and study design (PICOS) criteria. Studies were included if they met the following inclusion criteria: (1) the study population consisted of children or adolescents with overweight or obesity, with a mean age between 6 and under 18 years. Based on WHO growth standards, children and adolescents are classified as overweight if their BMI-for-age z-score (BMI-z) is ≥+1 and <+2 SD, and as obese if BMI-z is ≥+2 SD and their BMI is at or above the 85th percentile; (2) the exercise intervention involved high-intensity interval training (HIT) lasting for at least two weeks; and (3) “high-intensity training” was defined as all-out effort, maximal running speed (MAS), or an intensity of at least 90% of VO_2max_ [24], “85~95% maximal heart rate (HR_max_)” [25] or “≥100% maximum aerobic speed (MAS) [26]; (4) Outcomes were compared to those of a no-training control group (CON), a moderate-intensity training group (MIT—including interval and/or continuous training), or to pre-intervention data in single-arm studies; (5) outcomes included CRP, IL-6, and TNF-α; (6) study design included randomized and non-randomized controlled trials or pre–post single-arm trials; and (7)—accessible in English. The exclusion criteria were: (1) failure to retrieve a complete abstract or full text; (2) intervention inadequately described or did not fall within the scope of HIT; (3) repeated publication of literature; and (4) study protocols, observation/cross-sectional studies, animal-model studies, books, and reviews.

### 2.4. Data Extraction

After the studies were selected, two reviewers (MC and MXZ) independently carried out data extraction. They collected information on study characteristics—such as author, year, country, number of participants, gender, age range or mean, study duration, and setting—as well as exercise training protocol features, including modality, intensity, frequency, total time, and work–rest ratio. Additionally, they extracted outcome variables, namely the mean and standard deviation values for circulating inflammatory biomarkers before and after the training intervention.

### 2.5. Quality Assessment

The quality and risk of bias of the included studies were independently assessed by two reviewers (XDW and PS) using the Cochrane approach. For our risk of bias assessment, we used the Risk of Bias-2 (ROB-2) tool, which is designed for randomized studies [27], and ROBINS-I bias risk assessment for the non-randomized controlled study [28]. The risk-of-bias assessment covered several domains: bias stemming from the randomization process, bias due to departures from the planned interventions, bias related to incomplete outcome data, bias in how outcomes were measured, and bias in the choice of reported results. For each domain, judgments of “low risk,” “some concerns,” or “high risk” were assigned according to answers to a set of signaling questions. These evaluations were then combined to produce an overall risk-of-bias judgment for the study outcome under examination. The Cochrane Collaboration Risk of Bias Tool was used to assess the quality and risk of bias for multi-arm studies, and the NIH quality assessment tool for single-group trials. The results are summarized by the Review Manager software (version 5.3, http://tech.cochrane.org/revman, accessed on 12 January 2026) and presented by a traffic light diagram to provide a concise and intuitive summary. Details were provided in Supplementary Table S1.

### 2.6. Publication Bias

Publication bias was examined through both Egger’s and Begg’s statistical tests, with a significance threshold set at *p* ≤ 0.05 indicating potential bias [29]. However, it should be noted that Egger’s test becomes unreliable when fewer than ten studies are included in the analysis; therefore, emphasis was placed on visual inspection of funnel plots supplemented by Begg’s test. Funnel plots were generated for each meta-analysis to facilitate visual assessment, followed by Egger’s regression test to statistically verify the presence of publication bias. In cases where significant publication bias was detected, the robustness of the findings was further evaluated using the trim-and-fill method [30].

### 2.7. Statistical Analyses

The post-test mean values or change scores, together with their associated standard deviations (SDs), were analyzed using STATA software (V 15.0, StataCorp, College Station, TX, USA). Meta-analyses were performed to evaluate the impact of high-intensity interval training (HIT) on inflammatory biomarkers compared to moderate-intensity training (MIT), including both moderate-intensity interval training (MIIT) and moderate-intensity continuous training (MICT). Results from random-effects meta-analyses are presented in the figures, while mixed-effects model outcomes are described in the text. Effect sizes were reported as standardized mean differences (SMD), accompanied by 95% confidence intervals (CIs). A *p*-value of less than 0.05 was considered statistically significant. According to Cohen’s guidelines, effect sizes greater than 0.8 SMD were classified as large, between 0.5 and 0.8 as moderate, between 0.2 and 0.5 as small, and below 0.2 as negligible [31]. Heterogeneity was assessed using Cochrane’s Q test and the I^2^ statistic, with thresholds of <25%, 50%, and 75% indicating low, moderate, and high levels of heterogeneity, respectively. To explore whether intervention characteristics influenced the observed effects, we performed meta-regression and subgroup analyses based on unadjusted data. The factors examined included: (1) duration of the intervention (in weeks); (2) HIT volume (total training time), defined as the cumulative time spent on high-intensity intervals including recovery periods but excluding warm-up and cool-down phases; (3) the work-to-rest ratio of HIT, referring to the duration of exercise and recovery within each interval; and (4) HIT work time, which denotes the actual high-intensity portion of each interval.

## 3. Results

### 3.1. Literature Results

The search initially returned 339 articles from four databases. In total, 311 records were retained after excluding duplicates. The first screened by title and abstract, and then the eligible full texts were reviewed to ascertain whether they met the inclusion and exclusion criteria. Following the screening, 12 studies met the criteria and were retained, of which 1 study used single HIT arms without a control group [19], 5 studies included HIT and CON arms [20,22,32,33,34], and 6 studies included HIT and MIT arms [21,35,36,37,38,39] (Figure 1). Briefly, in this meta-analysis the mean ages of 659 participants ranging from 8 [39] to 16 [21] years old were included (Table 1). In terms of biological sex, two studies included only females [33,37], three studies included only males [19,34,36], and both males and females were included in the other studies [20,21,22,32,35,38,39]. The intervention duration among 15 studies ranged from 6 [19,34] to 48 [20] weeks; the intervention duration commonly used in most studies is 12 weeks. Of the included studies, two studies from China [19,22], two studies from UK [21,32], two studies from Iran [34,36], two studies from Brazil [35,39], and one study was conducted each in Tunisia [37], Serbia [33], Spain [20], and Colombia [39].

### 3.2. Risk of Bias

**Figure 2:**
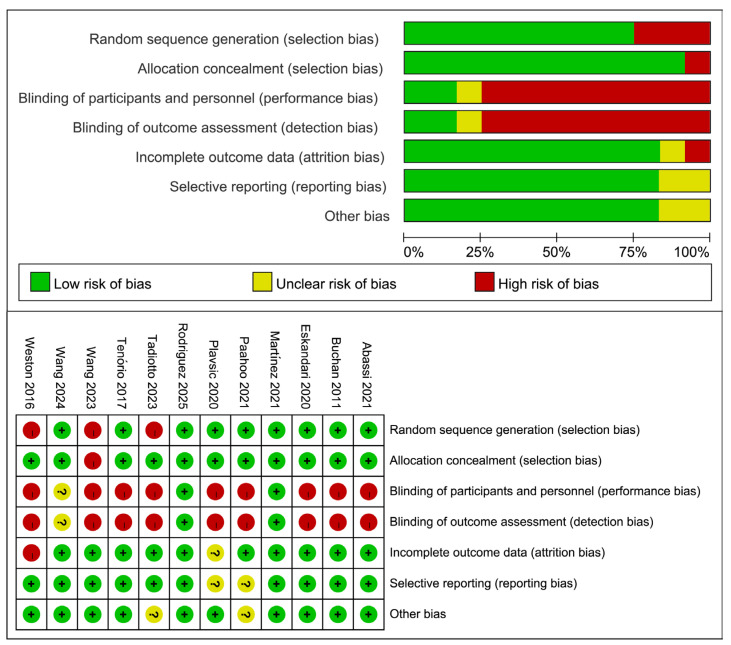
Evaluation of bias risk in the selected studies. Studies included: Abassi, 2021 [37]; Buchan, 2011 [21]; Eskandari, 2020 [34]; Martinez, 2021 [20]; Paahoo, 2021 [36]; Plavsic, 2021 [33]; Rodriguez, 2025 [39]; Tadiotto, 2023 [38]; Tenório, 2017 [35]; Wang, 2023 [19]; Wang, 2024 [22]; Weston, 2016 [25].

The results of the bias risk, based on the Cochrane Collaboration Risk of Bias Tool, in various quality domains are illustrated in Figure 2 and the Supplementary Table S1. Specifically, most trials showed low risks of bias across critical methodological areas—including random sequence generation, allocation concealment, handling of incomplete outcome data, selective outcome reporting, and other sources of bias. However, high risks emerged in relation to blinding of participants and personnel, along with outcome assessment procedures. Owing to the distinct characteristics of the exercise training intervention implemented in this study, careful deliberation is required when allocating participants. Unless two studies [20,39] explicitly described the blinding methods, all other studies were classified as having a high risk of bias. And we conducted a ROBINS-I bias risk assessment on the included non-randomized controlled study [20].

### 3.3. Meta-Analyses

**Figure 3:**
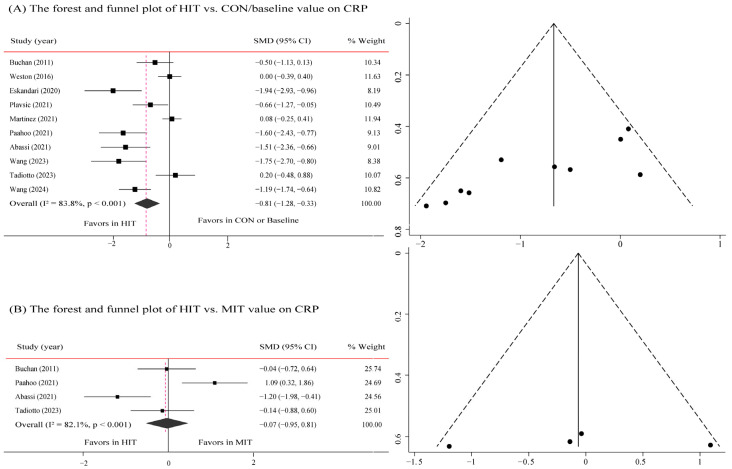
Forest and funnel plot of the effects of HIT on CRP. (**A**) The forest and funnel plot of HIT vs. CON/baseline value on CRP; (**B**) The forest and funnel plot of HIT vs. MIT value on CRP. Studies included: Buchan, 2011 [21]; Weston, 2016 [25]; Eskandari, 2020 [34]; Plavsic, 2021 [33]; Martinez, 2021 [20]; Paahoo, 2021 [36]; Abassi, 2021 [37]; Wang, 2023 [19]; Tadiotto, 2023 [38]; Wang, 2024 [22].

Pooled analyses of 10 studies showed that HIT resulted in a significant reduction in CRP with a large effect size (SMD: −0.81; 95% CI: −1.28 to −0.33; τ^2^ = 0.46, *p* = 0.001) (Figure 3A). However, the effect estimate should be interpreted with caution due to substantial heterogeneity across studies (τ^2^ = 0.38; I^2^ = 83.8%, *p* = 0.001). This heterogeneity indicates considerable uncertainty regarding the true magnitude of the effect. The Egger’s test (*p* = 0.003) and Begg’s test (*p* = 0.049) observed publication bias, but the assessment of the funnel plots did not observe publication bias. Subgroup analysis (Table 2) suggested a potential moderating effect of work-and-rest ratio ≥ 1 (SMD: −0.89; 95% CI: −1.62 to −0.17) and training time ≤ 20 min (SMD: −0.75; 95% CI: −1.33 to −0.18) of HIIT on reducing CRP level. However, the effects of HIIT on reducing CRP level may not be associated with intervention duration (<12 weeks or ≥12 weeks) and work time (>30 s or ≤30 s).

In addition, Pooled analyses of four studies showed that when compared with MIT, HIT was not superior in reducing CRP (SMD: −0.07; 95% CI: −0.95 to 0.81, τ^2^ = 0.66, *p* = 0.878). The assessment of the funnel plots did not observe publication bias. The results of Egger’s (*p* = 0.909) and Begg’s tests (*p* = 0.909) are consistent with it (Figure 3B).

**Figure 4:**
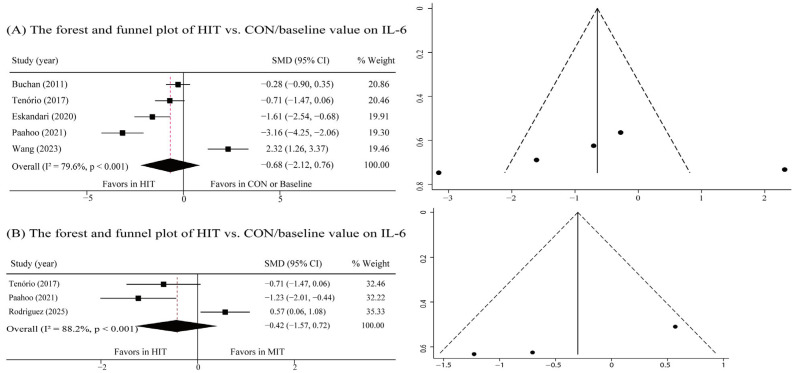
Forest and funnel plot of the effects of HIT on IL-6. (**A**) The forest and funnel plot of HIT vs. CON/baseline value on IL-6; (**B**) The forest and funnel plot of HIT vs. MIT value on IL-6. Studies included: Buchan, 2011 [21]; Tenório, 2017 [35]; Eskandari, 2020 [34]; Paahoo, 2021 [36]; Wang, 2023 [19]; Rodriguez, 2025 [39].

Meta-analysis of five studies indicated that HIT did not lead to a statistically significant reduction in IL-6 levels, despite a relatively large effect size (SMD: −0.63; 95% CI: −2.12 to 0.76; τ^2^ = 2.49, *p* = 0.354) (Figure 4A). Notably, there was considerable moderate heterogeneity among the included studies (I^2^ = 92.9%, *p* = 0.001). With the assessment of the funnel plots (visual plot revealed a roughly symmetrical distribution) and the Begg’s test (*p* = 0.824), no publication bias was observed. In addition, when compared with MIT, HIT was not superior on reducing IL-6 (SMD: −0.42; 95% CI: −1.57 to 0.72, τ^2^ = 0.89, *p* = 0.467) (Figure 4B). With the assessment of the funnel plots, no publication bias was observed, and the Begg’s test (*p* = 0.114) was confirmatory.

**Figure 5:**
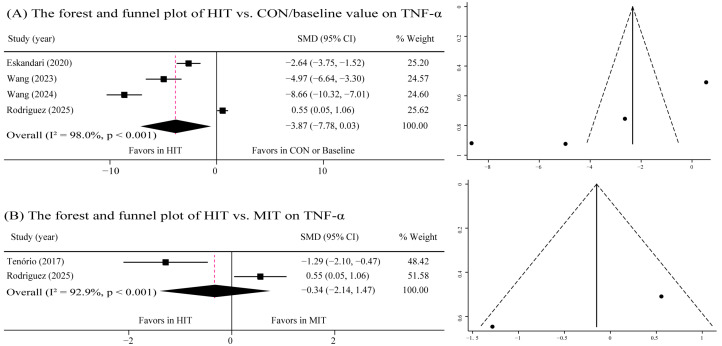
Forest and funnel plot of the effects of HIT on TNF-α. (**A**) The forest and funnel plot of HIT vs. CON/baseline value on TNF-α; (**B**) The forest and funnel plot of HIT vs. MIT value on TNF-α. Studies included: Eskandari, 2020 [34]; Wang, 2023 [19]; Wang, 2024 [22]; Rodriguez, 2025 [39]; Tenório, 2017 [35].

A meta-analysis pooling data from four studies yielded a combined SMD of −0.387 (95% CI: −7.78 to 0.03; τ^2^ = 15.40, *p* = 0.052), indicating that HIT was not effective in reducing TNF-α levels in children with obesity (Figure 5A). Visual inspection of the funnel plots suggested the presence of publication bias, which was further supported by a significant result from Begg’s test (*p* = 0.038). In addition, when compared with MIT, HIT was not superior on reducing TNF-α (SMD: −0.34; 95% CI: −2.14 to 1.47, τ^2^ = 1.57, *p* = 0.715) (Figure 5B). No publication bias was detected via funnel plot assessment. Egger’s test results could not be generated owing to the limited number of included studies (*n* = 2); whereas Begg’s test yielded non-significant findings (*p* = 1.000).

We did not conduct a subgroup analysis on the effect of HIT on IL-6 and TNF-α due to a lack of sufficient studies.

## 4. Discussions

This systematic review and meta-analysis of 12 RCTs and non-RCTs involving 659 children with obesity revealed that HIT significantly lowers CRP levels, but shows no significant effect on IL-6 and TNF-α. As the first meta-analysis to evaluate HIT’s influence on inflammatory cytokines in this population, subgroup analyses further identified that protocols with a work-to-rest ratio of at least 1:1 and sessions lasting 20 min or less were most effective for reducing CRP, aligning with findings from Cao et al. [13] Meanwhile, the results reinforce previous meta-analyses that investigated various health and disease conditions, confirming that HIT can reduce CRP levels [40], even in children with obesity. However, some studies have reported inconsistent results. For instance, a systematic review and meta-analysis indicated that exercise training did not significantly affect CRP levels in children and adolescents [41]. In addition, a meta-analysis conducted by García-Hermoso et al. suggests that the diverse range of exercise modalities—such as aerobic, resistance, and combined training—may lead to a lack of significant effect of physical activity on CRP levels in children who are overweight [42]. A study conducted by Zhao et al. found that HIT did not affect CRP levels in children with obesity. However, it is important to note that this study involved only a single HIT session, which may have introduced significant bias into the results [40]. This meta-analysis encompasses a large number of recently published trials and employs different analytical methods; the different modalities of exercise (aerobic exercise vs. HIT) and the different populations (adults vs. children) may contribute to the inconsistencies observed with prior studies.

There is well-established evidence demonstrating a physiological association between obesity and elevated levels of CRP [43]. Adipose tissue functions as an endocrine organ by secreting a variety of cytokines and hormones. Elevated levels of free fatty acids have been shown to correlate with increased concentrations of CRP [44]. Regular exercise training can reduce CRP levels through several interconnected mechanisms. A central pathway involves diminishing total body fat and visceral adipose tissue, which subsequently lowers the release of pro-inflammatory cytokines. Additionally, physical activity supports vascular health by enhancing endothelial function, boosts metabolic efficiency via improved insulin sensitivity, strengthens cellular defense systems through increased antioxidant capacity, and reshapes immune responses by modulating macrophage activation patterns [45]. Furthermore, controversy persists regarding the impact of HIT on inflammatory markers within obese cohorts, and the pathway through which HIT diminishes CRP concentrations remains inadequately characterized. An investigation led by Abassi et al. posits that the attenuation of CRP levels noted among obese adolescents post-HIT intervention may be attributable to concomitant decreases in adiposity and body mass [37]. In contrast, research by Weston and Buchan presents conflicting results, showing an increase in CRP levels in children after engaging in HIT [21,32]. However, existing evidence indicates that prolonged exercise training can enhance the functional capacity of skeletal muscles and immune cells, which in turn may help reduce systemic inflammatory markers [46]. Additionally, the gain in skeletal muscle mass associated with HIT could also play a role in lowering CRP levels and mitigating chronic inflammation [47]. The clinical relevance of HIT potentially lies in its ability to reduce CRP levels, a key factor given CRP’s established role as an independent predictor of cardiovascular disease in adults and a valuable tool for early diagnosis in pediatric populations [48,49].

Elevated concentrations of the pro-inflammatory cytokines IL-6 and TNF-α, commonly observed in obese children and adolescents, are associated with an increased likelihood of cardiometabolic disease [50]. Less is known about how exercise training affects pro-inflammatory biomarkers in children and adolescents. However, our findings are that HIT did not reduce IL-6 and TNF-α. This aligns with previous research on children and adolescents with overweight/obesity, which indicated no significant effects on IL-6 or TNF-α [51]. In contrast, Khalaf et al.’s meta-analysis focused on children and adolescents [17], as well as Chen et al.’s network meta-analysis concerning overweight and obese populations [52]; both demonstrated that exercise significantly reduces IL-6 and/or TNF-α. Consistent with our study, Zhao et al.’s meta-analysis found that four different training modalities (resistance training, aerobic training, aerobic plus resistance training, and HIT) did not reduce TNF levels in children with obesity [40]. Some studies indicate that the duration of the training period may be a crucial factor influencing the effectiveness of TNF-α intervention, at least a minimum of one year to achieve a significant reduction in TNF-α levels [53]. Additionally, evidence shows that exercise training reduces IL-6 and CRP in adolescents but not in younger children, and the magnitude of benefit depends on the type of exercise performed [17]. The observed differences may arise from variations in study design, sample size, participant characteristics, and inclusion criteria. For instance, children and adolescents may experience lower training effectiveness and completion rates. However, there are relatively few studies available; for example, the meta-analysis by Sirico et al. only included two related studies [54], while Han et al.’s meta-analysis incorporated data from four to five studies. This limited number of studies may result in insufficient strength for the meta-analyses.

The potential mechanism by which HIT reduces inflammatory cytokines remains unclear. It may involve a balance between inflammatory and anti-inflammatory cytokines, as well as a decrease in pro-inflammatory cells [55]. For example, IL-6 released during exercise can have anti-inflammatory effects, although it may also indicate a pro-inflammatory state under certain conditions [56]. In obese individuals, HIT may decrease the inflammatory status by repairing damaged endothelial cells and reducing visceral adipose tissue (VAT) [57]. Additionally, long-term exercise might lower levels of pro-inflammatory monocytes, contributing to improved TNF-α levels [58]. Elevated concentrations of pro-inflammatory cytokines in childhood obesity can heighten the risk of cardiovascular metabolic diseases, underscoring that even minor fluctuations in these biomarkers carry clinical significance [59]. The differences in age and developmental level of the subjects in this study may be potential influencing factors for HIT to improve IL-6 levels. Additionally, muscle injury has a significant impact on the response of inflammatory factors, such as IL-6. It was demonstrated that muscle damage and inflammatory responses vary with age and maturation, increasing as a function of pubertal development. Although both prepubertal and postpubertal boys generally exhibit minimal muscle damage, IL-6 responses increase markedly during puberty due to the greater muscle mass and enhanced cytokine release from contracting fibers, rather than injury, highlighting developmental differences in inflammatory reactivity [60]. In addition, the soluble IL-6 receptor (sIL-6R) is influenced not only by training load but also by psychological stress. In obese children, participation in structured sports combined with parental pressure may create psychological stress, which can alter sIL-6R responses and therefore influence IL-6 signaling [61]. In the future, it is essential to conduct relevant exercise intervention experiments and animal model trials to verify the effects and mechanisms of high-intensity training (HIT) on inflammatory biomarkers in children with obesity. Additionally, we must consider how psychological stress may influence inflammatory biomarkers cell pathways and other related factors.

This study demonstrated notable strengths, including a methodical literature search strategy that incorporated cutting-edge research and the application of meta-analysis to generate robust evidence and actionable guidance for advancing exercise training and weight management in children. Nevertheless, certain limitations should be noted. Firstly, considerable heterogeneity was observed across the pooled effect sizes, likely stemming from variations in methodology, study design, exercise regimens, and overall study quality. The application of the trim-and-fill method revealed no substantial publication bias, which supports the reliability of the results despite a potential threat to their robustness. A second consideration is that the relatively small sample sizes in some studies highlight the need for subsequent research involving larger, more diverse populations. Third, the insufficient number of included studies makes the fitting results of this study on the intervention effect of HIT on IL-6 and TNF-α not robust, and it is also impossible to further explore the influencing factors of HIT intervention through subgroup analysis. Measuring sIL-6R and sgp130 alongside IL-6 is essential for distinguishing whether IL-6 exerts predominantly pro- or anti-inflammatory effects, as highlighted by Febbraio [62]. Potential exercise effects on sgp130 were not assessed in many studies. Given that exercise can modify both IL-6 and sIL-6R, it is important to consider that sgp130 may also be affected, thereby altering the balance between classical and trans-signaling pathways and influencing the inflammatory profile of the response [63]. Genetic variability in IL-6 signaling was not accounted for. The rs2228145 SNP, which substantially affects IL-6 trans-signaling, remains underexplored in sports science despite its relevance for inflammatory responses and individual differences in training adaptation.

## 5. Conclusions

Our research provides support for high-intensity interval training as an efficient approach to lowering CRP in children with obesity. The positive effects on inflammatory cytokines are influenced by various components of the HIT protocol, including the duration of the intervention, the work-to-rest ratio, the duration of each work bout, and the overall training time per session.

## Figures and Tables

**Table 1:** Summary of study characteristics.

Author Year Country	N (Sex)	Weight Status	Age (Years)	BMI (kg/m^2^) /BMI-z	Intervention Characteristics			Outcome
					Group (*n*)	Exercise Protocol	Duration (Fre d/w)	
Buchan [21] 2011 UK	57 (B and G)	OW	16.4 ± 0.7	22.2 ± 2.7	HIT (17)	Running 4~6 × 30 s (all-out): 30 s (0)	7 weeks (3)	IL-6, CRP
					MIT (16)	Running (70% VO_2max_) with 20 min		
Weston [32] 2016 UK	52 (B and G)	OW/OB	14.1 ± 0.3	21.8 ± 4.5	HIT (41)	Multiple exercise with 4 × [45 s (≥ 90% HR_max_): 90 s (0)]	10 weeks (3)	CRP
Tenório [35] 2017 Brazil	31 (B and G)	OB	15.0 ± 1.0	33.1 ± 4.4	HIT (31)	Running NR (350 kcal per session)	12 weeks (3)	IL-6, TNF-α
					MIT (31)	Running NR (350 kcal per session)		
Plavsic [33] 2020 Serbia	44 (G)	OB	15.8 ± 1.4	32.6 ± 2.7	HIT (22)	Running 4 × 4 min (85~90% HR_max_): 3 min (70% HR_max_)	12 weeks (3)	CRP
Eskandari [34] 2020 Iran	24 (B)	OB	15.4 ± 1.1	32.0 ± 2.2	HIT (13)	Jump rope 7~20 × 1~4 min (60~90/min): 30 s (0)	6 weeks (3)	IL-6, TNF-α, CRP
Martínez [20] 2021 Spain	143 (B and G)	OW/OB	10.0 ± 0.7	18.3 ± 3.8	HIT (77)	Game 4 × 240 s (85~90% HR_max_): 180 s (65~75% HR_max_)	48 weeks (4)	CRP
Paahoo [36] 2021 Iran	45 (B)	OW/OB	11.0 ± 1.0	25.3 ± 1.0	HIT (15)	Running 3 × 10 × [10 s (100~110% MAS):10 s (50%MAS)]: 3–4 min (0)	12 weeks (2)	IL-6, CRP
				25.0 ± 0.7	MIT (15)	Running (40~70% HRR) with 30 min		
Abassi [37] 2021 Tunisia	43 (G)	OW/OB	NR	32.6 ± 3.6	HIT (15)	Running 2 × 6 × [30 s (100~110% MAS): 30 s (50% MAS)]: 4 min (0)	12 weeks (3)	CRP
				33.1 ± 5.6	MIT (15)	Running 2 × 6 × [30 s (70~80% MAS): 30 s (50% MAS)]: 4 min (0)		
Tadiotto [38] 2023 Brazil	52 (B and G)	OW	11–16	2.4 ± 1.0 (BMI-z)	HIT (13)	Cycling with 3 × 4 × [30 s (80~100% HRR): 60 s (NR)]: 4 min (0)	12 weeks (3)	CRP
				2.0 ± 0.8 (BMI-z)	MIT (15)	Cycling (35~75%HRR) with 60 min		
Wang [19] 2023 China	15 (B)	OW	12.9 ± 0.8	20.6 ± 3.0	HIT (6)	Cycling 4~6 × 30 s (7.5% BW): 30 s (25 w)	6 weeks (3)	IL-6, TNF-α, CRP
Wang [22] 2024 China	60 (B and G)	OW	8.3 ± 1.0	19.5 ± 1.9	HIT (30)	Runing and Cycling 5 × 4 min (90% VO_2max_): 2 min (0)	12 weeks (3)	CRP, TNF-α
Rodriguez [39] 2025 Colombia	62 (B and G)	OW/OB	8.1 ± 0.1	NR	HIT (6)	Game 4 × 6 min (85~95% HR_max_): 2 min (0)	16 weeks (3)	IL-6, TNF-α
				NR	MIT (6)	Aerobic 3 × 10 min (65% HR_max_): 5 min (0)		

**Abbreviations:** B, boys; BW, body weight; CON, control group; d/w, day per week; Fre, frequency; G, girls; HIT, high-intensity interval training; IL-6, interleukin-6; MIT, moderate-intensity continuous and/or interval training; HR_max/peak_, maximal or peak heart rate; HRR, heart rate reserve; CRP, C-reactive protein; MAS, maximum aerobic speed; NR, not-reported; OB, obesity; OW, overweight; TNF-α, tumor necrosis factor-alpha.

**Table 2:** The effect of HIT on CRP in a subgroup of children with obesity.

Outcomes	Pooled/Total (%)	SMD (95% CI)	Favored in HIT		Favored in CON/Pre	*I*^2^ (%)	*p*-Value
Duration			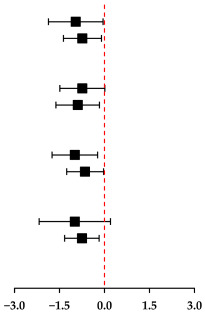				
<12	4/10 (40)	−0.96 (−1.87, −0.05)				85.7	0.001
≥12	6/10 (60)	−0.74 (−1.37, −0.10)				85.5	0.001
Work-and-rest ratio							
≥1	5/10 (50)	−0.74 (−1.49, 0.01)				86.8	0.001
<1	5/10 (50)	−0.89 (−1.62, −0.17)				83.9	0.001
Work time							
>30 s	5/10 (50)	−0.99 (−1.75, −0.23)				79.3	0.001
≤30 s	5/10 (50)	−0.65 (−1.26, −0.03)				86.5	0.001
Total time							
>20 min	3/10 (30)	−0.99 (−2.18, 0.20)				89.1	0.001
≤20 min	7/10 (70)	−0.75 (−1.33, −0.18)				83.8	0.001
	
**Outcomes**	**Pooled/Total (%)**	**SMD (95% CI)**	**Favored in** **HIT**	**Favored in** **MIT**		***I*** **^2^** **(%)**	***p*** **-Value**
Duration			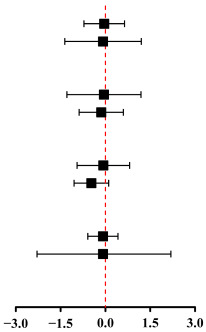				
<12	1/4 (25)	−0.04 (−0.72, 0.64)				0.00	-
≥12	3/4 (75)	−0.08 (−1.36, 1.20)				88.1	0.001
Work-and-rest ratio							
≥1	3/4 (75)	−0.05 (−1.29, 1.19)				88.0	0.001
<1	1/4 (25)	−0.14 (−0.88, 0.60)				0.00	-
Work time							
>30 s	4/4 (100)	−0.07 (−0.95, 0.81)				94.3	0.001
≤30 s	0/4 (0)	-				-	-
Total time							
>20 min	2/4 (50)	−0.08 (−0.59, 0.42)				0.00	0.845
≤20 min	2/4 (50)	−0.05 (−2.29, 2.19)				94.0	0.001
	

## Data Availability

The original contributions presented in this study are included in the article/Supplementary Materials. Further inquiries can be directed to the corresponding author.

## Supplementary Materials

The following supporting information can be downloaded at: https://www.mdpi.com/article/10.3390/metabo16010088/s1, Methods 1: Electronic Search Strategy; Methods 2: Excluded Studies and Reasons for Exclusion; Figure S1: The trim and fill of HIT vs. CON on CRP; Figures S2–S5: Subgroup analysis of HIT vs. CON on CRP in moderator variables; Figures S6–S9: Subgroup analysis of HIT vs. CON on IL-6 in moderator variables; Table S1: Risk of bias assessment of included studies.

## Abbreviations

Listed below are the abbreviations used throughout this manuscript:

BW	Body weight
CON	Control group
CRP	C-reactive protein
HIT	High-intensity interval training
HR_max/peak_	Maximal or peak heart rate
HRR	Heart rate reserve
IL-6	Interleukin-6
MAS	Maximum aerobic speed
MIT	Moderate-intensity training including continuous training and interval training
TNF-α	Tumor necrosis factor-alpha

## Appendix A

**Table A1:** Electronic Search Strategy from 4 databases.

Database	Search Details	Results
PubMed	((((((((((“high-intensity interval training”) OR (“high-intensity intermittent training”)) OR (“high-intensity interval exercise”)) OR (“high-intensity interval”)) OR (“high-intensity intermittent”)) OR (HIIT)) OR (“interval training”)) OR (“sprint interval”))) AND (child * OR kid OR boy OR girl * OR adolescent * OR preadolescent * OR youth OR student * OR teenage * OR adolescence)) AND (CRP OR “C-reactive protein” OR “Tumor necrosis factor-alpha” OR TNF-α OR Interleukin OR IL-6 OR inflammatory OR inflammation)	192
Web of Science	TS = (“high-intensity interval training” OR “high-intensity intermittent training” OR “high-intensity interval exercise” OR “high-intensity intermittent exercise” OR “aerobic interval training” OR HIIT) AND TS = (child OR children OR adolescen * OR student OR boy * OR girl * OR youth OR teenage *) AND TS = (IL-6 OR “Interleukin” OR CRP OR “C-reactive protein” OR inflammatory OR inflammation OR “Tumor necrosis factor-alpha” OR “TNF-α”)	43
Scopus	TITLE-ABS-KEY (overweight OR obesity OR obese) AND TITLE-ABS-KEY (child * OR kid OR boy OR girl * OR adolescent * OR preadolescent * OR youth OR student * OR teenage * OR adolescence) AND TITLE-ABS-KEY (IL-6 OR “Interleukin” OR CRP OR “C-reactive protein” OR inflammatory OR “Tumor necrosis factor-alpha” OR “TNF-α”) AND TITLE-ABS-KEY (HIIT OR “high-intensity interval training” OR “high-intensity intermittent training” OR “high-intensity interval exercise” OR “high-intensity interval” OR “high-intensity intermittent” OR “interval training” OR “sprint interval”)	44
Embase	‘body weight’/exp or ‘overweight’/exp or ‘obesity’/exp AND ‘high-intensity interval training’/exp or ‘high-intensity intermittent training’/exp or ‘high-intensity interval exercise’/exp or ‘high-intensity intermittent exercise’/exp or ‘aerobic interval training’/exp or ‘hiit’/exp or ‘sprint interval’/exp AND ‘child *’/exp or ‘adolescent’/exp or ‘boy *’/exp or ‘girl *’/exp or ‘kid *’/exp or ‘youth’/exp AND ‘crp’/exp or ‘c-reactive protein’/exp or ‘Interleukin’/exp or ‘IL-6′/exp or ‘inflammatory’/exp or ‘inflammation’/exp or ‘Tumor necrosis factor-alpha’/exp or ‘TNF-α’/exp	60
