# Supplementary material for: The Effects of High-Intensity Interval Training on Inflammatory Cytokines in Children and Adolescents with Obesity: A Systematic Review and Meta-Analysis
Source: Metabolites. 2026 Jan 21;16(1):88. doi: 10.3390/metabo16010088 (PMC12844070; doi:10.3390/metabo16010088)
Supplement: Supplementary file 1 [file metabolites-16-00088-s001.zip › metabolites-4032012-supplementary.pdf]

Supplementary Files

Methods 1. Electronic Search Strategy

Methods 2. Excluded Studies and Reasons for Exclusion

Table S1. Risk of bias assessment of included studies (n=9) examining the effect of HIIT in children with obesity.

Figure S1. The trim and fill of HIT vs CON on CRP

Figure S**2.** Subgroup analysis of HIT vs CON on CRP in moderator variable '**duration**'. 0 means < 12-week, 1 means ≥ 12-week.

Figure S3. Subgroup analysis of HIT vs CON on CRP in moderator variable '**Work and rest ratio**'. 0 means = 1, 1 means < 1, 2 means > 1.

Figure S**4.** Subgroup analysis of HIT vs CON on CRP in moderator variable '**Work time**'. 0 means > 30-s, 1 means ≤ 30-s.

Figure S**5.** Subgroup analysis of HIT vs CON on CRP in moderator variable '**total time**'. 0 means > 20-min, 1 means ≤ 20-min.

Figure S6. Subgroup analysis of HIT vs CON on IL-6 in moderator variable **'duration'**. 0 means < 12 week, 1 means ≥ 12 week.

Figure S7. Subgroup analysis of HIT vs CON on IL-6 in moderator variable **'Work and rest ratio'.** 0 means = 1, 1 means < 1, 2 means > 1.

Figure S8. Subgroup analysis of HIT vs CON on IL-6 in moderator variable **'Work time'.** 0 means > 30-s, 1 means ≤ 30-s.

Figure S9. Subgroup analysis of HIT vs CON on IL-6 in moderator variable **'total time'.** 0 means > 20-min, 1 means ≤ 20-min.

This supplementary material has been provided by the authors to give readers additional information about their work.

Methods 1. Electronic Search Strategy from 4 databases

PubMed (192 results)

((((((((("high intensity interval training") OR ("high intensity intermittent training")) OR ("high intensity interval exercise")) OR ("high intensity interval")) OR ("high intensity intermittent")) OR (HIIT)) OR ("interval training")) OR ("sprint interval"))) AND (child* OR kid OR boy OR girl* OR adolescent* OR preadolescent* OR youth OR student* OR teenage* OR adolescence)) AND (CRP OR "C-reactive protein" OR "Tumor necrosis factor-alpha" OR TNF-α OR Interleukin OR IL-6 OR inflammatory OR inflammation)

Web of Science (43 results)

TS=("high intensity interval training" OR "high intensity intermittent training" OR "high intensity interval exercise" OR "high intensity intermittent exercise" OR "aerobic interval training" OR HIIT) AND TS=(child OR children OR adolescen* OR student OR boy* OR girl* OR youth OR teenage*) AND TS=(IL-6 OR "Interleukin" OR CRP OR "C-reactive protein" OR inflammatory OR inflammation OR "Tumor necrosis factor-alpha" OR "TNF-α")

Scopus (44 results)

TITLE-ABS-KEY (overweight OR obesity OR obese) AND TITLE-ABS-KEY (child* OR kid OR boy OR girl* OR adolescent* OR preadolescent* OR youth OR student* OR teenage* OR adolescence) AND TITLE-ABS-KEY (IL-6 OR "Interleukin" OR CRP OR "C-reactive protein" OR inflammatory OR "Tumor necrosis factor-alpha" OR "TNF-α") AND TITLE-ABS-KEY (HIIT OR "high intensity interval training" OR "high intensity intermittent training" OR "high intensity interval exercise" OR "high intensity interval" OR "high intensity intermittent" OR "interval training" OR "sprint interval")

Embase (60 results)

'body weight'/exp or 'overweight'/exp or 'obesity'/exp AND 'high intensity interval training'/exp or 'high intensity intermittent training'/exp or 'high intensity interval exercise'/exp or 'high intensity intermittent exercise'/exp or 'aerobic interval training'/exp or 'hiit'/exp or 'sprint interval'/exp AND 'child*'/exp or 'adolescent'/exp or 'boy*'/exp or 'girl*'/exp or 'kid*'/exp or 'youth'/exp AND 'crp'/exp or 'c-reactive protein'/exp or 'Interleukin'/exp or 'IL-6'/exp or 'inflammatory'/exp or 'inflammation'/exp or 'Tumor necrosis factor-alpha'/exp or 'TNF-α'/exp

Methods 2. Excluded Studies and Reasons for Exclusion

Reason for exclusion: Duplicated data (7 results)

Wang X., Fan M., Meng Q., Zhang D., Liu T. (2024). Effects of high-intensity interval training on lipid metabolism, tumor necrosis factor-α, and C-reactive proteinin overweight children. American Journal of Translational Research, 16(11), 6889-6902. http://dx.doi.org/10.62347/LZIS9354

Tadiotto M.C., Corazza P.R.P., Menezes-Junior F.J., Moraes-Junior F.B., Tozo T.A.A., Purim K.S.M., Mota J., Leite N. (2023). Effects and individual response of continuous and interval training on adiponectin concentration, cardiometabolic risk factors, and physical fitness in overweight adolescents. European Journal of Pediatrics, 182(6), 2881-2889. http://dx.doi.org/10.1007/s00431-023-04974-6

Abassi W., Ouerghi N., Nikolaidis P.T., Hill L., Racil G., Knechtle B., Feki M., Bouassida A. (2022). Interval Training with Different Intensities in Overweight/Obese Adolescent Females. International journal of sports medicine, 43(5), 434-443. http://dx.doi.org/10.1055/a-1648-4653

Paahoo A., Tadibi V., Behpoor N. (2021). Effectiveness of Continuous Aerobic Versus High-Intensity Interval Training on Atherosclerotic and Inflammatory Markers in Boys With Overweight/Obesity. Pediatric exercise science, 33(3), 132-138. http://dx.doi.org/10.1123/pes.2020-0138

van Biljon A., McKune A.J., DuBose K.D., Kolanisi U., Semple S.J. (2018). Do Short-Term Exercise Interventions Improve Cardiometabolic Risk Factors in Children?. Journal of Pediatrics, 203, 325-329. http://dx.doi.org/10.1016/j.jpeds.2018.07.067

Weston K.L., Azevedo L.B., Bock S., Weston M., George K.P., Batterham A.M. (2016). Effect of novel, school-based High-intensity Interval Training (HIT) on cardiometabolic health in adolescents: Project FFAB (Fun Fast Activity Blasts) - An exploratory controlled before-and-after trial. PLoS ONE, 11(8). http://dx.doi.org/10.1371/journal.pone.0159116

Supriya R., Delfan M., Saeidi A., Samaie S.S., Al Kiyumi M.H., Escobar K.A., Laher I., Heinrich K.M., Weiss K., Knechtle B., Zouhal H. (2023). Spirulina Supplementation with High-Intensity Interval Training Decreases Adipokines Levels and Cardiovascular Risk Factors in Men with Obesity. Nutrients, 15(23). http://dx.doi.org/10.3390/nu15234891

Reason for exclusion: No information on the outcome of interest (9 results)

Sun F., Williams C.A., Sun Q., Hu F., Zhang T. (2024). Effect of eight-week high-intensity interval training versus moderate-intensity continuous training programme on body composition, cardiometabolic risk factors in sedentary adolescents. Frontiers in Physiology, 15. http://dx.doi.org/10.3389/fphys.2024.1450341

Gomes Torres A.C.M.B., Leite N., de Souza R.L.R., Pizzi J., Milano-Gai G.E., Lazarotto L., Tureck L.V., Furtado-Alle L. (2024). Variants in inflammation-related genes influence the outcomes of physical exercise programs: A longitudinal study in Brazilian adolescents with overweight and obesity. Genetics and Molecular Biology, 47(4). http://dx.doi.org/10.1590/1678-4685-GMB-2023-0211

Personalised Exercise Training in Adolescents With Type 2 Diabetes for the Early Prevention of Beta Cell Dysfunction: Development of a New Algorithm to Integrate Clinical, Epigenomic and Machine Learning Models (2022), Identifier: NCT05344768. http://clinicaltrials.gov/study/NCT05344768

Soltani N., Esmaeil N., Marandi S.M., Hovsepian V., Momen T., Shahsanai A., Kelishadi R. (2020). Assessment of the Effect of Short-Term Combined High-Intensity Interval Training on TLR4, NF-κB and IRF3 Expression in Young Overweight and Obese Girls. Public Health Genomics, 23(1-2), 26-36. http://dx.doi.org/10.1159/000506057

Tenorio T.R.S., Balagopal P.B., Andersen L.B., Ritti-Dias R.M., Hill J.O., Lofrano-Prado M.C., Prado W.L. (2018). Effect of Low- Versus High-Intensity Exercise Training on Biomarkers of Inflammation and Endothelial Dysfunction in Adolescents With Obesity: A 6-Month Randomized Exercise Intervention Study. Pediatric exercise science, 30(1), 96-105. http://dx.doi.org/10.1123/pes.2017-0067

Alizadeh H., Safarzade A. (2019). High intensity intermittent training induces anti-inflammatory cytokine responses and improves body composition in overweight adolescent boys. Hormone Molecular Biology and Clinical Investigation, 39(3). http://dx.doi.org/10.1515/hmbci-2019-0004

Racil G., Aouichaoui C., Hawani A., Signorelli P., Chamari K., Migliaccio G.M., Trabelsi Y., Padulo J. (2024). The impact of interval training on adiponectin to leptin ratios and on blood pressures in severely obese adolescent girls: A randomized controlled trial. Journal of sports sciences, 1-9. http://dx.doi.org/10.1080/02640414.2024.2369447

Salus M., Tillmann V., Remmel L., Unt E., Maestu E., Parm U., Magi A., Tali M., Jurimae J. (2022). Effect of Sprint Interval Training on Cardiometabolic Biomarkers

and Adipokine Levels in Adolescent Boys with Obesity. International Journal of Environmental Research and Public Health, 19(19). http://dx.doi.org/10.3390/ijerph191912672

Pedro HN, AE Morano, Ricardo RA, André O W (2025). Cytokine and adipokine response following high-intensity interval running and cycling in female adolescents. Eur J Appl Physiol. 19. http://dx.doi.org/10.1007/s00421-025-05851-w.

Reason for exclusion: Inappropriate intervention (1 results)

Deng H., Chen Y., Xing J., Zhang N., Xu L. (2024). Systematic low-grade chronic inflammation and intrinsic mechanisms in polycystic ovary syndrome. Frontiers in Immunology, 15. http://dx.doi.org/10.3389/fimmu.2024.1470283

Reason for exclusion: Inappropriate study design (20 results)

Zhou Y., Wang H. (2025). Exercise intervention for obese children and adolescents. Zhongguo Ertong Baojian Zazhi, 33(2), 127-131. http://dx.doi.org/10.11852/zgetbjzz2024-1457

Mehta P., Pattnaik S., Bhowmik S., Saha J., Goel N. (2024). Commentary on “breathing breakthrough: Muscle training improves lung function in juvenile arthritis”. Pediatric Pulmonology, 59(11), 2729-2730. http://dx.doi.org/10.1002/ppul.27149

Khalafi M., Symonds M.E., Faramarzi M., Sharifmoradi K., Maleki A.H., Rosenkranz S.K. (2024). The effects of exercise training on inflammatory markers in children and adolescents: A systematic review and meta-analysis. Physiology and Behavior, 278. http://dx.doi.org/10.1016/j.physbeh.2024.114524

Shantsila E., Choi E.-K., Lane D.A., Joung B., Lip G.Y.H. (2024). Atrial fibrillation: comorbidities, lifestyle, and patient factors. The Lancet Regional Health - Europe, 37. http://dx.doi.org/10.1016/j.lanepe.2023.100784

Wang A., Zhang H., Liu J., Yan Z., Sun Y., Su W., Yu J.-G., Mi J., Zhao L. (2023). Targeted Lipidomics and Inflammation Response to Six Weeks of Sprint Interval Training in Male Adolescents. International Journal of Environmental Research and Public Health, 20(4). http://dx.doi.org/10.3390/ijerph20043329

Zhao H., Cheng R., Teng J., Song G., Huang C., Yuan S., Lu Y., Shen S., Liu J., Liu C. (2022). A Meta-Analysis of the Effects of Different Training Modalities on the

Inflammatory Response in Adolescents with Obesity. International Journal of Environmental Research and Public Health, 19(20). http://dx.doi.org/10.3390/ijerph192013224

Kim S.-H., Cho Y.-H., Kim H.-Y. (2022). Distinctive clinical features of spontaneous pneumoperitoneum in neonates: A retrospective analysis. World Journal of Clinical Cases, 10(23), 8124-8132. http://dx.doi.org/10.12998/wjcc.v10.i23.8124

"HIIT Med Kiloene" Investigating the Physical, Metabolic, and Psychosocial Effects of High-intensity Interval Training (HIIT) in Childhood Obesity (2022), Identifier: NCT05465057. http://clinicaltrials.gov/study/NCT05465057

Efecto de Los Juegos Infantiles de Alta Intensidad Comparado Con Los de Mediana Intensidad Sobre la regulación autonómica Cardiaca, Biomarcadores de Obesidad y composición Corporal en Niños y Niñas de 6 a 9 años Con Obesidad o Sobrepeso (2021), Identifier: NCT05294601. http://clinicaltrials.gov/study/NCT05294601

Falqueto H., Junior J.L.R., Silverio M.N.O., Farias J.C.H., Schoenfeld B.J., Manfredi L.H. (2021). Can conditions of skeletal muscle loss be improved by combining exercise with anabolic–androgenic steroids? A systematic review and meta-analysis of testosterone-based interventions. Reviews in Endocrine and Metabolic Disorders, 22(2), 161-178. http://dx.doi.org/10.1007/s11154-021-09634-4

Khalafi M., Symonds M.E. (2021). The impact of high intensity interval training on liver fat content in overweight or obese adults: A meta-analysis. Physiology and Behavior, 236. http://dx.doi.org/10.1016/j.physbeh.2021.113416

Tomczyk M.M., Dolinsky V.W. (2020). The cardiac lipidome in models of cardiovascular disease. Metabolites, 10(6), 1-19. http://dx.doi.org/10.3390/metabo10060254

Yang J., Li Y., Wen Z., Liu W., Meng L., Huang H. (2021). Oscillospira - a candidate for the next-generation probiotics. Gut Microbes, 13(1). http://dx.doi.org/10.1080/19490976.2021.1987783

Headid R.J., Park S.-Y. (2021). The impacts of exercise on pediatric obesity. Clinical and Experimental Pediatrics, 64(5), 196-207. http://dx.doi.org/10.3345/CEP.2020.00997

Ouerghi N., Ben Fradj M.K., Bezrati I., Feki M., Kaabachi N., Bouassida A. (2017). Effect of High-Intensity Interval Training on Plasma Omentin-1 Concentration in Overweight/Obese and Normal-Weight Youth. Obesity Facts, 10(4), 323-331. http://dx.doi.org/10.1159/000471882

Gorzi A., Rahmani A., Mohammadi Z., Neto W.K. (2021). Effects of different lengths of high-intensity interval training microcycles on the systemic and hippocampal inflammatory state and antioxidant balance of immature rats. Molecular Biology Reports, 48(6), 5003-5011. http://dx.doi.org/10.1007/s11033-021-06484-w

Sindhwani R., Bora K.S., Hazra S. (2025). The dual challenge of diabesity: pathophysiology, management, and future directions. Naunyn-Schmiedeberg's Archives of Pharmacology, 398(5), 4891-4912. http://dx.doi.org/10.1007/s00210-024-03713-4

Ariadel-Cobo D.G., Estebanez B., Gonzalez-Arnaiz E., Garcia-Perez M.P., Rivera-Viloria M., Pintor de la Maza B., Barajas-Galindo D.E., Garcia-Sastre D., Ballesteros-Pomar M.D., Cuevas M.J. (2025). Influence of Klotho Protein Levels in Obesity and Sarcopenia: A Systematic Review. International Journal of Molecular Sciences, 26(5). http://dx.doi.org/10.3390/ijms26051915

Moritz C.E.J., Vieira A.F., de Melo-Marins D., Figueiro F., Battastini A.M.O., Reischak-Oliveira A. (2022). Effects of physical exercise on the functionality of human nucleotidases: A systematic review. Physiological Reports, 10(18). http://dx.doi.org/10.14814/phy2.15464

Moslemi E., Dehghan P., Khani M. (2022). The effect of date seed (Phoenix dactylifera) supplementation on inflammation, oxidative stress biomarkers, and performance in active people: A blinded randomized controlled trial protocol. Contemporary Clinical Trials Communications, 28. http://dx.doi.org/10.1016/j.conctc.2022.100951

Reason for exclusion: Inappropriate population (14 results)

Hu S., Liu X., Ding Y., Chen J., Wang X. (2025). Effects of exercise and walnut oil on CES1 and inflammatory factors in the liver of type 2 diabetic rats. European Journal of Medical Research, 30(1). http://dx.doi.org/10.1186/s40001-025-02377-x

Soltani N., Esmaeil N., Marandi S.M., Hovsepian V., Momen T., Shahsanai A. (2023). A 2-week combined high-intensity interval training regulates inflammatory status in young females with obesity. Science and Sports, 38(2), 174-181. http://dx.doi.org/10.1016/j.scispo.2021.12.006

Effect of L-citrulline and HIIT on Arterial Stiffness, Body Composition, and Lipid Profile in Adolescents with Steatosis Associated with Metabolic Dysfunction (MASLD) (2023), Identifier: NCT05778266.

http://clinicaltrials.gov/study/NCT05778266

Vella C.A., Taylor K., Drummer D. (2017). High-intensity interval and moderate-intensity continuous training elicit similar enjoyment and adherence levels in overweight and obese adults. European journal of sport science, 17(9), 1203-1211. http://dx.doi.org/10.1080/17461391.2017.1359679

Leggate M., Carter W.G., Evans M.J.C., Vennard R.A., Sribala-Sundaram S., Nimmo M.A. (2012). Determination of inflammatory and prominent proteomic changes in plasma and adipose tissue after high-intensity intermittent training in overweight and obese males. Journal of Applied Physiology, 112(8), 1353-1360. http://dx.doi.org/10.1152/japplphysiol.01080.2011

Yang M.-Y., Chen H.-Y., Ho C.-H., Huang W.-C. (2025). Impact of Probiotic Supplementation and High-Intensity Interval Training on Primary Dysmenorrhea: A Double-Blind, Randomized Controlled Trial Investigating Inflammation and Hormonal Modulation. Nutrients, 17(4). http://dx.doi.org/10.3390/nu17040622

Ahmed A.S., Ahmed M.S. (2023). The impact of high intensity interval training on serum omentin-1 levels, lipid profile, and insulin resistance in obese men with type 2 diabetes mellitus. Isokinetics and Exercise Science, 31(3), 221-231. http://dx.doi.org/10.3233/IES-220117

Paolucci E.M., Loukov D., Bowdish D.M.E., Heisz J.J. (2018). Exercise reduces depression and inflammation but intensity matters. Biological Psychology, 133, 79-84. http://dx.doi.org/10.1016/j.biopsycho.2018.01.015

Nguyen T., Obeid J., Ploeger H.E., Takken T., Pedder L., Timmons B.W. (2012). Inflammatory and growth factor response to continuous and intermittent exercise in youth with cystic fibrosis. Journal of Cystic Fibrosis, 11(2), 108-118. http://dx.doi.org/10.1016/j.jcf.2011.10.001

Adair D., Hider A., Filbrun A.G., Tapley C., Bouma S., Iwanicki C., Nasr S.Z. (2022). Assessing the Utility of an Outpatient Exercise Program for Children With Cystic Fibrosis: A Quality Improvement Project. Frontiers in Pediatrics, 9. http://dx.doi.org/10.3389/fped.2021.734292

Rhibi F., Abderrahman A.B., Prioux J., Clark C.C.T., Bideau B., Besbes S., Hackney A.C., Granacher U., Zouhal H. (2022). Effects of different training intensities in high-intensity interval training (HIIT) on maximal aerobic velocity, hematological and muscle-damage markers in healthy young adults. BMC Sports Science, Medicine and Rehabilitation, 14(1). http://dx.doi.org/10.1186/s13102-022-00550-x

Soori R., Goodarzvand F., Akbarnejad A., Effatpanah M., Ramezankhani A., Teixeira A.L., Ghram A. (2020). Effect of high-intensity interval training on clinical and laboratory parameters of adolescents with attention deficit hyperactivity disorder. Science and Sports, 35(4), 207-215. http://dx.doi.org/10.1016/j.scispo.2019.08.002

Huang W.-C., Chiu P.C., Ho C.H. (2022). The Sprint-Interval Exercise Using a Spinning Bike Improves Physical Fitness and Ameliorates Primary Dysmenorrhea Symptoms Through Hormone and Inflammation Modulations: A Randomized Controlled Trial. Journal of sports science & medicine, 21(4), 595-607. http://dx.doi.org/10.52082/jssm.2022.595

Haghighi A.H., Hajinia M., Askari R., Abbasian S., Goldfied G. (2022). Effect of high-intensity interval training and high-intensity resistance training on irisin and fibroblast growth factor 21 in men with overweight and obesity. Canadian Journal of Physiology and Pharmacology, 100(9), 937-944. http://dx.doi.org/10.1139/cjpp-2021-0712

Reason for exclusion: Exercise combined with other therapy, such as nutritional supplement, drug, etc. (1 results)

Sarkar S., Dey S.K., Datta G., Bandyopadhyay A. (2023). Vitamin C and E supplementation and high intensity interval training induced changes in lipid profile and haematological variables of young males. Sports Medicine and Health Science, 5(2), 137-145. http://dx.doi.org/10.1016/j.smhs.2023.03.006

Table S1. Risk of bias assessment of included studies (n=15) examining the effect of HIIT in children with obesity.

Study	Year	Random sequence generation (selection bias)	Allocation concealment (selection bias)	Blinding of patients and personnel (performance bias)	Blinding of outcome assessment (detection bias)	Incomplete outcome data (attrition bias)	Selective outcome reporting (reporting bias)	Any other bias
Buchan	2011	Low	Low	High	High	Low	Low	Low
Weston	2016	High	Low	High	High	Low	Low	Low
Tenório	2017	Low	Low	High	High	Low	Low	Low
Plavsic	2020	Low	Low	High	High	Unclear	Unclear	Low
Eskandari	2020	Low	Low	High	High	Low	Low	Low
Martínez	2021	Low	Low	Low	Low	Low	Low	Low
Paahoo	2021	Low	Low	High	High	Low	Unclear	Unclear
Abassi	2021	Low	Low	High	High	Low	Low	Low
Tadiotto	2023	High	Low	High	High	Low	Low	Unclear
Wang	2023	High	High	High	High	Low	Low	Low
Wang	2024	Low	Low	High	High	High	Unclear	Low
Rodriguez	2025	Low	Low	Low	Low	Low	Low	Low

See the Figure for summary information. It is not possible to truly blind patients to treatment allocation in exercise training trials; thus, this was not included in the overall risk of bias assessment of each study. Studies included: Buchan, 2011 [21]; Weston, 2016 [25]; Tenório, 2023 [35]; Plavsic, 2021 [33]; Eskandari, 2020 [34]; Martínez, 2021 [20]; Paahoo, 2021 [36]; Abassi, 2021 [37]; Tadiotto, 2023 [38]; Wang, 2023 [19]; Wang, 2024 [22]; Rodriguez, 2025 [39].

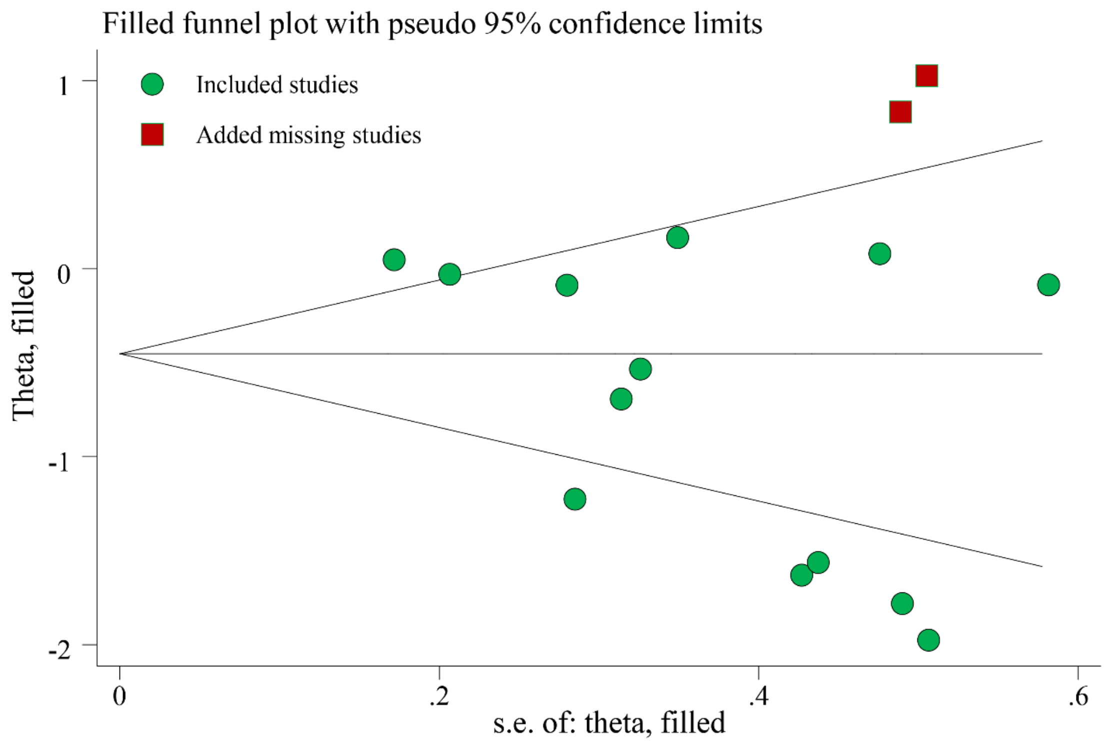

Figure S1. The trim and fill of HIT vs CON on CRP

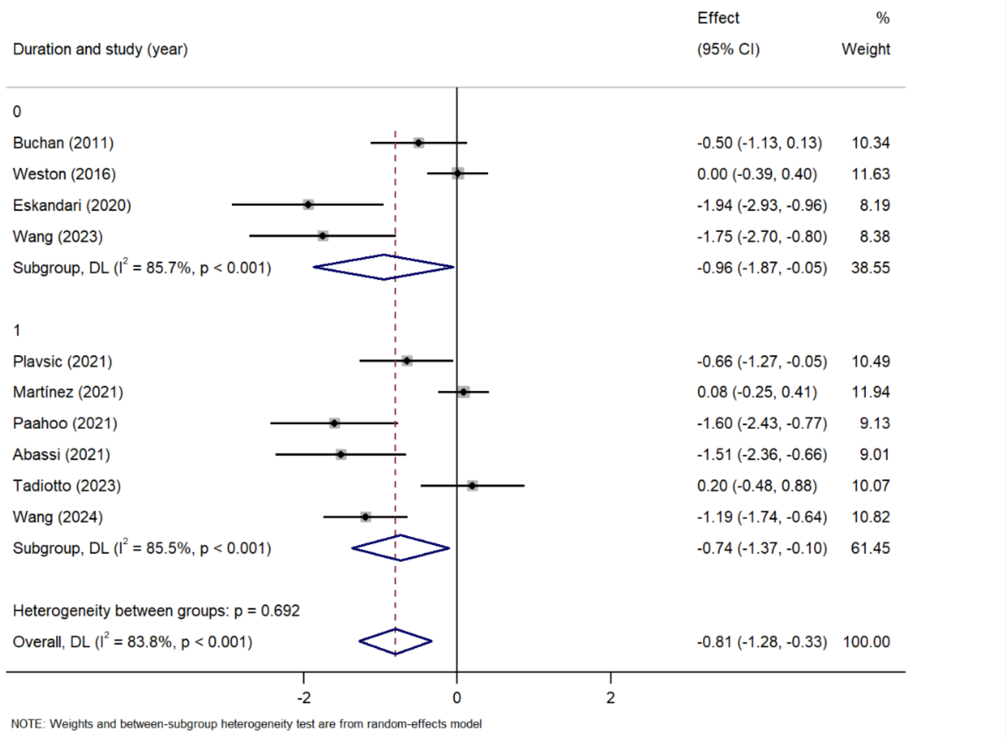

Figure S2. Subgroup analysis of HIT vs CON on CRP in moderator variable '**duration**'. 0 means < 12 week, 1 means ≥ 12 week. Studies included: Buchan, 2011 [21]; Weston, 2016 [25]; Eskandari, 2020 [34]; Tenório, 2023 [35]; Wang, 2023 [19]; Plavsic, 2021 [33]; Martínez, 2021 [20]; Paahoo, 2021 [36]; Abassi, 2021 [37]; Tadiotto, 2023 [38]; Wang, 2024 [22].

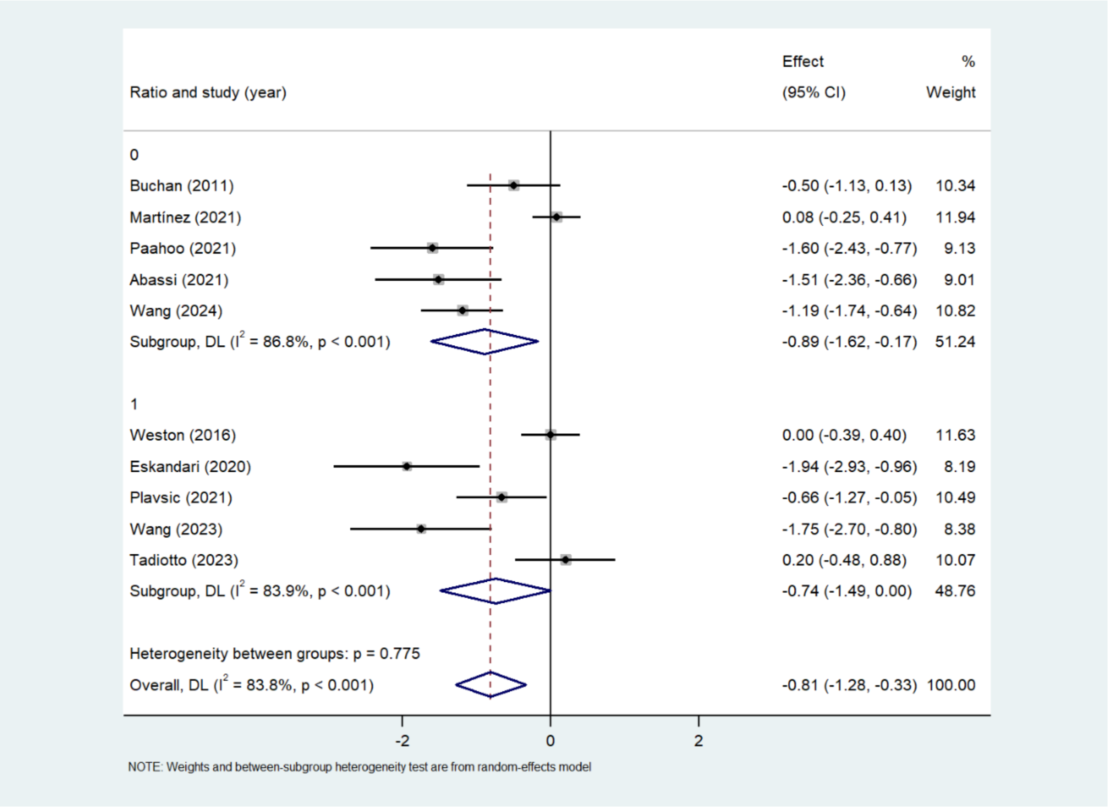

Figure S3. Subgroup analysis of HIT vs CON on CRP in moderator variable '**Work and rest ratio**'. 0 means = 1, 1 means < 1, 2 means > 1. Studies included: Buchan, 2011 [21]; Weston, 2016 [25]; Eskandari, 2020 [34]; Tenório, 2023 [35]; Wang, 2023 [19]; Plavsic, 2021 [33]; Martínez, 2021 [20]; Paahoo, 2021 [36]; Abassi, 2021 [37]; Tadiotto, 2023 [38]; Wang, 2024 [22].

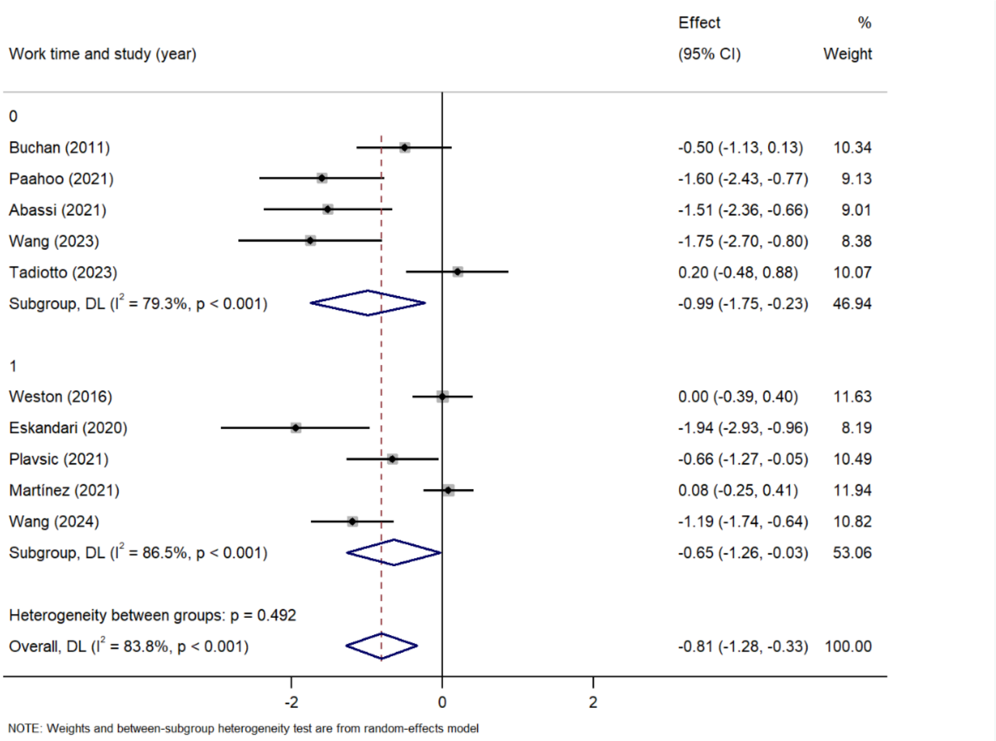

Figure S4. Subgroup analysis of HIT vs CON on CRP in moderator variable '**Work time**'. 0

means > 30-s, 1 means ≤ 30-s. Studies included: Buchan, 2011 [21]; Weston, 2016 [25]; Eskandari, 2020 [34]; Tenório, 2023 [35]; Wang, 2023 [19]; Plavsic, 2021 [33]; Martínez, 2021 [20]; Paahoo, 2021 [36]; Abassi, 2021 [37]; Tadiotto, 2023 [38]; Wang, 2024 [22].

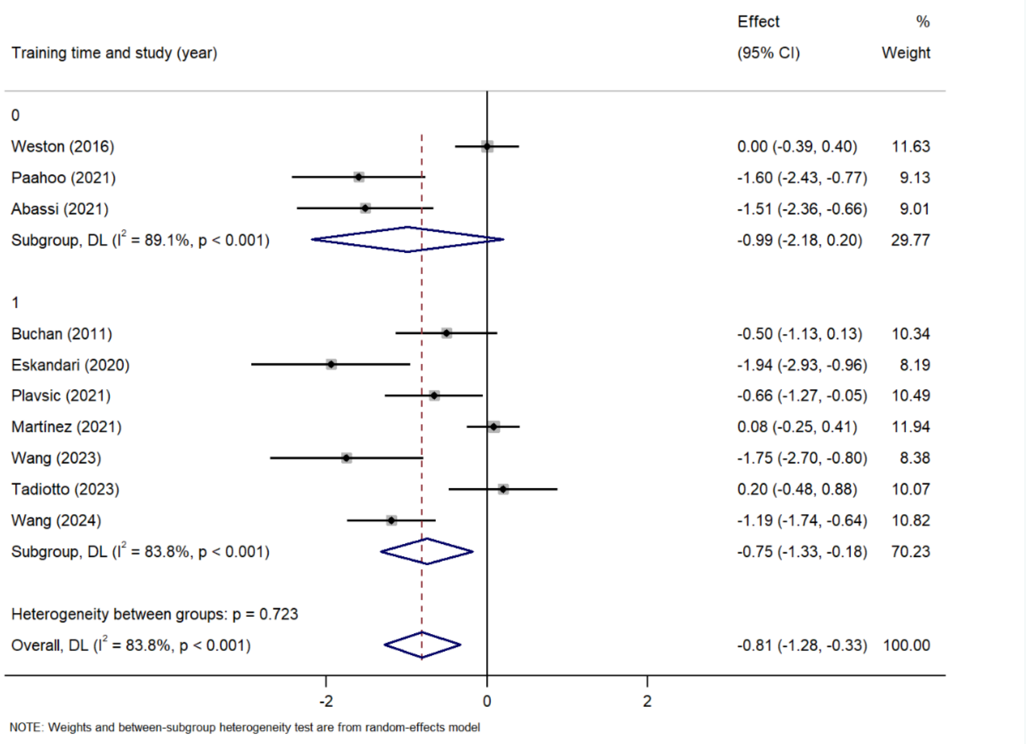

Figure S5. Subgroup analysis of HIT vs CON on CRP in moderator variable '**total time**'. 0 means > 20-min, 1 means ≤ 20-min. Studies included: Buchan, 2011 [21]; Weston, 2016 [25]; Eskandari, 2020 [34]; Tenório, 2023 [35]; Wang, 2023 [19]; Plavsic, 2021 [33]; Martínez, 2021 [20]; Paahoo, 2021 [36]; Abassi, 2021 [37]; Tadiotto, 2023 [38]; Wang, 2024 [22].

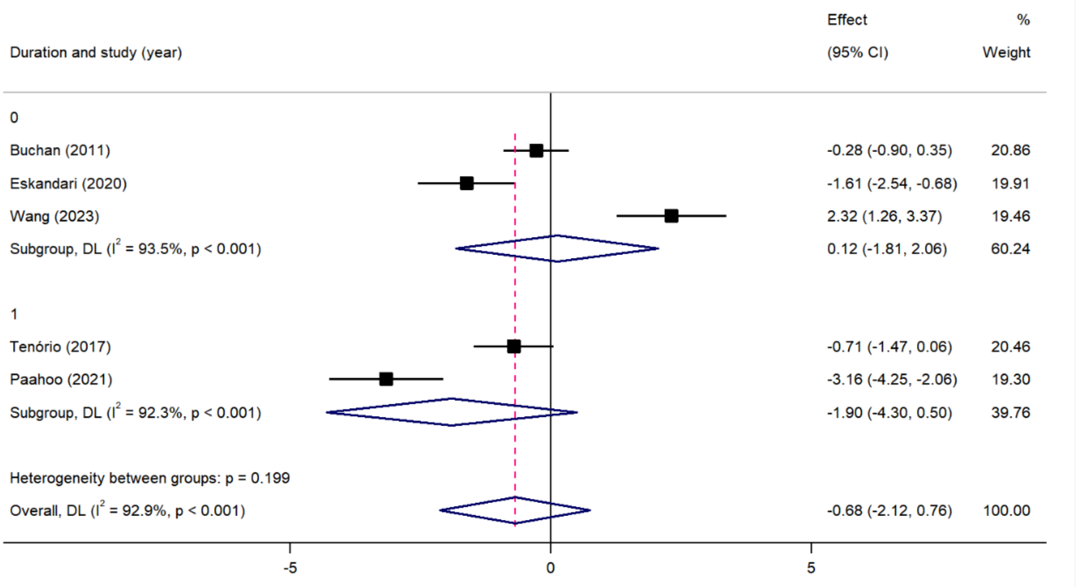

Figure S6. Subgroup analysis of HIT vs CON on IL-6 in moderator variable ‘duration’. 0 means < 12 week, 1 means ≥ 12 week. Studies included: Buchan, 2011 [21]; Eskandari, 2020 [34]; Wang, 2023 [19]; Tenório, 2023 [35]; Paahoo, 2021 [36].

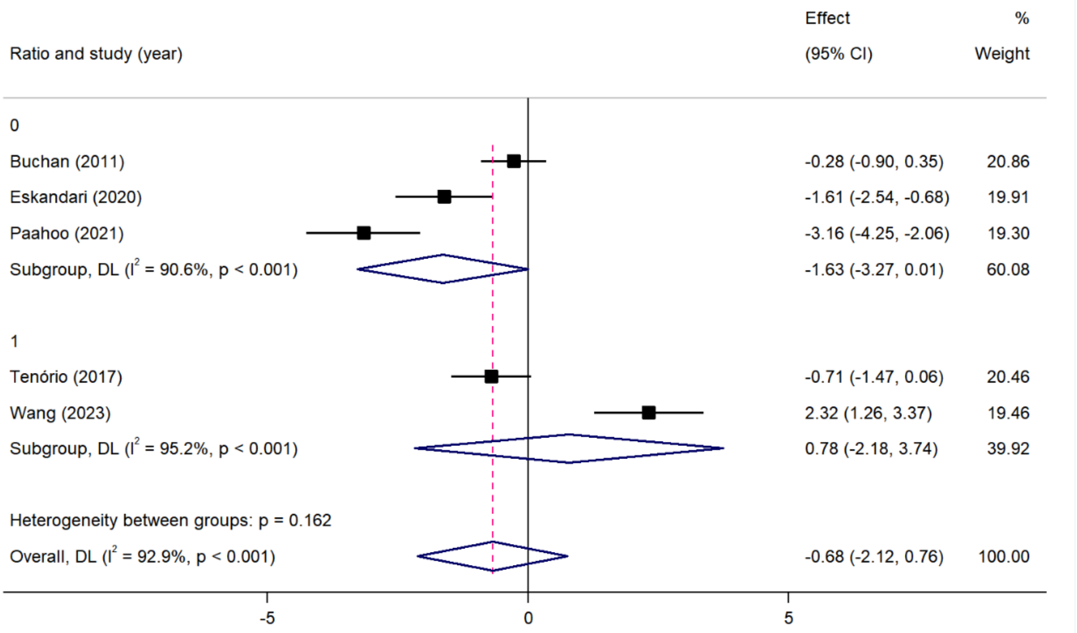

Figure S7. Subgroup analysis of HIT vs CON on IL-6 in moderator variable ‘Work and rest ratio’. 0 means = 1, 1 means < 1, 2 means > 1. Studies included: Buchan, 2011 [21]; Eskandari, 2020 [34]; Wang, 2023 [19]; Tenório, 2023 [35]; Paahoo, 2021 [36].

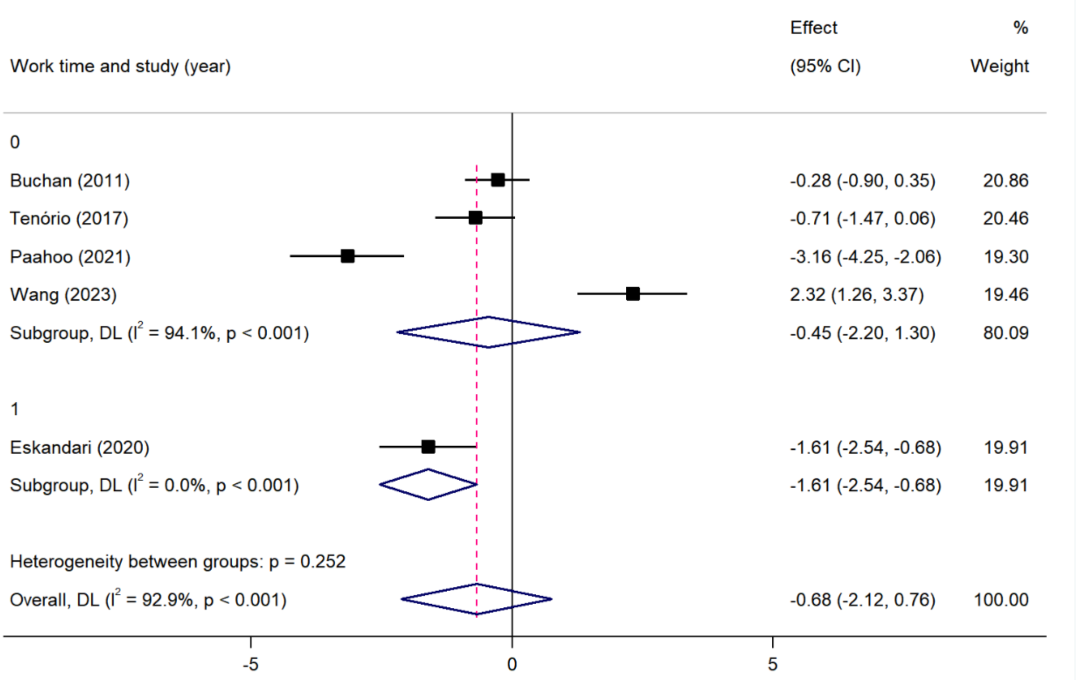

Figure S8. Subgroup analysis of HIT vs CON on IL-6 in moderator variable ‘Work time’. 0 means > 30-s, 1 means ≤ 30-s. Studies included: Buchan, 2011 [21]; Eskandari, 2020 [34]; Wang, 2023 [19]; Tenório, 2023 [35]; Paahoo, 2021 [36].

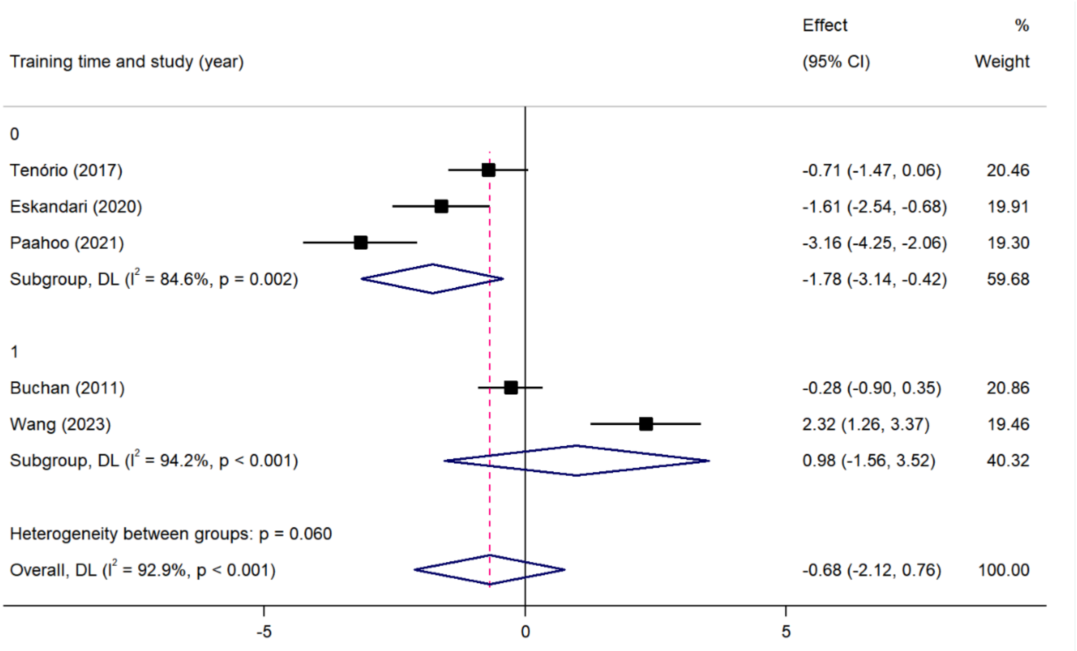

Figure S9. Subgroup analysis of HIT vs CON on IL-6 in moderator variable ‘total time’. 0 means > 20-min, 1 means ≤ 20-min. Studies included: Buchan, 2011 [21]; Eskandari, 2020 [34]; Wang, 2023 [19]; Tenório, 2023 [35]; Paahoo, 2021 [36].
